# Does Pregabalin Have Neuropsychotropic Effects?: A Short Perspective

**DOI:** 10.4306/pi.2009.6.2.55

**Published:** 2009-06-30

**Authors:** David M. Marks, Ashwin A. Patkar, Prakash S. Masand, Chi-Un Pae

**Affiliations:** 1 Department of Psychiatry and Behavioral Sciences, Duke University Medical Center, Duke Clinical Research Institute, Durham, NC, USA.; 2 Department of Psychiatry, The Catholic University of Korea College of Medicine, Seoul, Korea.

**Keywords:** Pregabalin, Analgesic, Anxiolytic, Anticonvulsant, Psychiatric disorders

## Abstract

Pregabalin is a newly developed synthetic gamma-aminobutyric acid (GABA) that is approved for the treatment of fibromyalgia and several neuropathy. It has been proven to show analgesic, anxiolytic, anticonvulsant and sleep enhancement effects, which could be applicable in the treatment of a variety of psychiatric disorders. There have been consistent reports that unexplained somatic symptoms (i.e., pain) may be a part of psychiatric disorders such as major depressive disorder (MDD) and anxiety disorders. Previous researches have also suggested the possible therapeutic potential of anticonvulsants as augmentation therapy or monotherapy in the treatment of mood disorders and anxiety disorders. Hence this short perspective tries to prompt and facilitate a shifting of researchers' attention to potential neuropsychotropic drug role of pregabalin to treat a wide range of neuropsychiatric disorders.

## Introduction

Pregabalin is a new synthetic molecule and a structural derivative of the inhibitory neurotransmitter gamma-aminobutyric acid (GABA). Pregabalin does not bind directly to GABA_A_, GABA_B_, or benzodiazepine receptors. In addition it does not augment GABA_A_ responses in cultured neurons, alter rat brain GABA concentration or have acute effects on GABA uptake or degradation. However, it was found that prolonged application of pregabalin increases the density of GABA transporter protein as well as the rate of functional GABA transport in cultured neurons. Pregabaline has no involvement with serotonin and dopamine receptors and does not inhibit dopamine, serotonin, or noradrenaline reuptake. It has no effects on sodium channels, opiate receptors, and cyclooxygenase enzyme activity.

Pregabalin is characterized by its potent high-affinity binding to the α2-δ subunit on voltage-gated calcium channels,^1^,^2^ and is approved for management of neuropathic pain associated with diabetic peripheral neuropathy and postherpetic neuralgia, adjunctive therapy for adult patients with partial onset seizures and management of fibromyalgia by the United States Food and Drug Administration (U.S. FDA).^3^-^6^

The drug may have a strong potential as one of promising neuropsychotropic agents in the monotherapy or augmentation treatment of various neuropsychiatric disorders based on its currently available preclinical and clinical data. Hence this short perspective will review the potential neuropsychotropic effects of pregabalin.

## Preclinical Evidence

The recent study of brief treatment with alprazolam initiated immediately after exposure to a traumatizing experience revealed that treatment effectively curbed short-term anxiety-like behaviors, but had no prevention value and caused a statistically significant increase in individual vulnerability to a trauma-reminder and even more so to re-exposure to the index stressor.^7^ In addition, benzodiazepines have been found not to be associated with the significant treatment effects on trauma-related anxiety symptoms. However, pregabalin was found to be valuable in the alleviation of anxiety symptoms associated with traumatic insults without a tendency of vulnerability to subsequent stress in a preclinical study.^7^

Pregabalin was also excellent in sleep efficiency as showing an enhancement of slow-wave sleep, reduction of the number of midnight awakenings, rapid eye movement sleep and sleep-onset latency, which are commonly seen in depression, anxiety and pain disorders.^8^ In addition, pregabalin demonstrated comparable to placebo in cognitive and psychomotor measures, while it was significantly superior to alprazolam.^9^

Pregabalin is a GABA analogue without abuse potential, although the action mechanism is not same with benzodiazepine agents. This property is important in patients who need to withdraw benzodiazepine or may have abuse propensity. Although the proper principal mechanism of pregabalin's effect on psychiatric disorders may not be fully explained, the modulation of calcium channels by which reduction of excitatory neurotransmitters release should account for its usefulness for various psychiatric disorders.^2^ It is in line with one of the action mechanisms of tricyclic antidepressants (TCAs) that have proven effectiveness in the treatment of depression, anxiety and pain diseases.

Several line of preclinical data have also suggested the neuroprotective effect of pregabalin by anti-apoptotic and anti-inflammatory actions, which is useful in protection of cognitive function in patients with depression and geriatric psychiatric population.^10^ The effects of the long-term exposure to pregabalin on neuronal damage and epileptogenesis induced by lithium-pilocarpine induced status epilepticus in which the effects of pregabalin was assessed in hippocampus and piriform and entorhinal cortices in rat brain sections. In the study, pregabalin induced neuroprotection in layer II of piriform cortex and layers III-IV of ventral entorhinal cortex of adult rats.^11^ The potential neuroprotective effect of pregabalin may add another optional treatment in the area of brain injury, cognitive decline in affective disorders, neurodegenerative disease and dementias.

## Clinical Evidence

As stated, pregabalin has been U.S. FDA-approved medication for fibromyalgia, which is interesting since fibromyalgia and major depressive disorder (MDD) share some commonalities. Numerous studies have found that fibromyalgia patients are at increased risk for a lifetime history of MDD as compared to normal community samples.^12^ Also, patients with fibromyalgia and MDD share symptomatologic similarities. Depressed mood, poor expression of emotion, decreased energy level, anergia, fatigue, easy upset to psychosocial stressors, aberration of sleep architectures and multiple vague somatic symptoms.^13^ Furthermore, the number of tender points in patients with fibromyalgia has been found to be correlated with depressive symptoms such as depressed mood, fatigue, disability, pain, and somatic symptoms as well as being connected with functional impairments.^14^

Recently an emerging evidence of potential role of pregabalin in the treatment of psychiatric disorders such as generalized anxiety disorder (GAD) and social anxiety disorder (SAD) has been available. The potential anti-depressive and anti-anxiety effects of pregabalin have been established in a number of randomized, placebo-controlled clinical trials (RCTs).^1^,^15^-^18^ In the first RCT of pregabalin for GAD,^15^ the mean baseline-to-endpoint decreases in total Hamilton Anxiety Rating Scale (HARS) score in patients with 600 mg/d of pregabalin (-44.4%) were significantly greater than the decrease in those given placebo (-29.7%), while it was not significantly different comparing with lorazepam. This trend was observed as early as the week 1 continuing till the end of the study, which was replicated in a subsequent similarly designed 4-week RCT: pregabalin was significantly superior over placebo (mean difference=3.9, p=0.001) as measured by HARS.^17^ The superiority of pregabalin along with rapid improvement over placebo was consistently demonstrated in other RCTs, indicating that pregabalin may be clearly useful and has definite clinical evidence for GAD.^1^,^19^ Interestingly, pregabalin 400 or 600 mg/d was compared with venlafaxine 75 mg/d, or placebo for 6-week (n=421). Pregabalin at both dosages was found be equivalent to the effect of venlafaxine in improvement of HARS with significant differences comparing with placebo.^20^ Aforementioned findings ultimately suggest that pregabalin has similar and comparable efficacy with contemporary anti-depressant and benzodiazepines as well as having partly favorable adverse events profile compared to benzodiazepines in the treatment of GAD. Although it was not robust as much as seen in GAD trials, pregabalin 600 mg/d was also effective in the treatment of SAD.^16^ In line with such RCTs, the effect size (ES) of pregabalin (ES=0.5) for GAD treatment was found be higher than contemporary antidepressants, serotonin-norepinephrine reuptake inhibitors (SNRIs)(ES=0.42) and selective serotonin reuptake inhibitors (SSRIs)(ES=0.36), and bezodiazepines (ES=0.38).^21^

According a post-hoc analysis of the existing clinical trial database, pregabalin at doses of 150 mg/d (32.1%), 300-450 mg/d (40.1%) and 600 mg/d (36.7%) was associated with statistically significant improvement in endpoint Hamilton Depression Rating Scale-17 item (HAMD-17) scores compared to placebo (22.8%).^18^ Moreover, pregabalin retained superior efficacy even in patients with more prominent depressive symptoms compared to placebo, especially demonstrating the most beneficial response with pregabalin 300-450 mg/d. This finding may indicate the potential of pregabalin as augmentation therapy for patients with treatment resistance, partially responding to current antidepressants or with comorbid anxiety disorders when subsequent RCTs replicate similar results.

Pregabalin was also effective in the treatment of benzodiazepine withdrawal symptoms in even chronic abusers.^22^-^24^ This issue is in particular important since many psychiatric patients as well as those seen in general practice have been easily exposed to abuse or dependence, which may lead to comorbid psychiatric and medical conditions as well as increment of individual and public health care costs. Hence pregabalin may be quite helpful when it could be applicable to the biological detoxification programs.

In the author's clinical experience, pregabalin was very useful as augmentation therapy in the treatment of posttraumatic stress disorder (PTSD) patients who were partially responsive to current antidepressants as well.^25^ The action mode of pregabalin, that is, a reduction of excitatory neurotransmitters such as glutamate fits well with major symptoms of PTSD, i.e., hypervigilance and hyperarousal.^26^

## Future Direction and Conclusion

**FIGURE 1:**
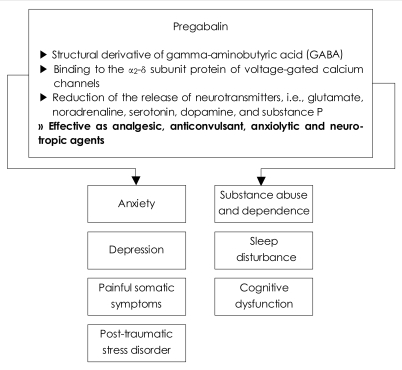
Pharmacological characteristics and potential usefulness of pregabalin in psychiatric disorders.

We may expect a substantial role of pregabalin for patients with depression, anxiety disorders, substance abuse and dependence, aggression and violence, interictal psychosis, sleep disturbance, unexplained somatic complaints (predominantly widespread pain), PTSD, neurodegenerative diseases and bipolar affective disorders (Figure 1).

Future researches should include whether or not pregabalin has early improvement effect in depression or anxiety disorders or any treatment effects as augmentation therapy for such patients who did not respond to conventional treatment options. Predictor analysis on response to pregabalin will be intriguing well. Given methodological pitfalls in currently available data, additional works are also needed to determine whether these favorable findings for pregabalin hold true over the long-term period, and whether patients having several comorbid psychiatric disorders are also responsive to pregabalin. Direct comparison studies will be also necessary to confirm differential outcomes between pregabalin and contemporary treatment regimes.
